# The influence of different digital registration methods on measurement accuracy of clear aligner treatment outcomes

**DOI:** 10.1186/s12903-025-07494-x

**Published:** 2025-12-26

**Authors:** Samar M. Adel, Nadia El-Harouni, Hassan E. Kassem, Abbas R. Zaher, Nikhillesh Vaiid

**Affiliations:** 1 Department of Orthodontics, Faculty of Dentistry, Alexandria University, Champollion Street, El Azarita, Alexandria, Egypt; 2 Department of Orthodontics, Saveetha Dental College, Saveetha Insitute of Medical and Technical Sciences, Only Orthodontics, Ground Floor, New Blue Gardenia Housing Society, Peddar Road, Mumbai, 400026 India

**Keywords:** Artificial intelligence, Registration, 3D digital models, 3D tooth movement, Digital orthodontics, Clear aligner therapy, Scanning

## Abstract

**Objectives:**

To compare the agreement of three digital model registration methods of different software packages in measuring angular and linear tooth movements obtained with Clear Aligner Treatment Therapy.

**Methods:**

Thirty-two maxillary and mandibular intraoral pre-treatment (T1) and progress (T2) scans of patients undergoing clear aligner therapy were randomly selected, converted to STL files and exported to Geomagic, OrthoAnalyzer, and Compare model registration software packages. The amount of tooth movement of all maxillary and mandibular teeth was calculated in six degrees of freedom.

**Results:**

The angular and linear change in tooth position between T1 and T2 was compared using three different digital model registration software packages. Continuous data was expressed as mean and standard deviation. Intra class Correlation Coefficient for agreement between software programs was used. Significance of the obtained results was expressed at *p* ≤ 0.01. Differences larger than 0.5 mm for linear measurements and 2º for angular measurements were considered clinically relevant. Geomagic and Compare demonstrated excellent consistency in most dimensions (ICC > 0.90), including maxillary tip (ICC = 0.929) and mandibular tip (ICC = 0.912); however, combinations involving OrthoAnalyzer showed the poorest performance in mandibular torque measurements (ICC = 0.484–0.493), with OrthoAnalyzer’s mean values (2.72 ± 4.30°) significantly higher than the other two software platforms; in the OG direction, all combinations involving OrthoAnalyzer showed ICC < 0.5, indicating ‘poor reliability.

**Conclusions:**

Geomagic and Compare software demonstrated excellent consistency in measuring tooth movements, whereas OrthoAnalyzer consistently showed poor reliability for mandibular torque (ICC < 0.5) and occlusogingival movements (ICC < 0.5). These differences exceeded the clinical thresholds (0.5 mm for translations, 2° for angular changes) and highlight the importance of cautious interpretation when comparing results across platforms.

**Clinical significance:**

The use of CAT is exponentially growing in Orthodontics. Studies that compare treatment accuracy by superimposing two digital models, generate values based on a digital registration software. Does the software employed; cause a difference to the values generated, and subsequently our interpretation of accuracy of a given system? This study aims to address this knowledge gap.

## Introduction

Orthodontics has witnessed immense technological advancements in the last decade. Three-dimensional imaging has expanded diagnostic and treatment planning options, scanners now provide an alternative to conventional impressions, and digital models are replacing stone models for treatment planning, simulations, measurements and appliance fabrication. The increasing patient demand for esthetically pleasing treatment options combined with the drive towards personalized treatment, have given rise to a noticeably increasing number of clear aligner patients and systems that now serve as alternatives to conventional fixed appliance systems [1–6].

The efficacy and predictability of various tooth movements in clear aligner therapy (CAT) has been studied by digital superimposition of achieved and predicted or planned outcomes by various software superimposition methods [7–12]. Through this appraisal of tooth movement, the professional can understand the capabilities and limitations of therapeutics and the mechanical principles employed [13–16]. The outcome of these studies has inspired newer materials and attachment geometries that have enhanced appliance predictability of aligners [9, 17].

For quantifying treatment effects, variable techniques and software packages have been utilized for 3D digital model registration and tooth movement measurements. They differ in the registration methods they offer, in the method of measuring 3D tooth movements, costs, as well as in the time and complexity to perform a specific task [18–20]. Most of them use computer-based registration algorithms in combination with operator input [21–27].

Algorithms available for 3D registration include either iterative closest point algorithms (registration based on the whole surface area to bring the two models to best fit each other), or best-fit registration of arbitrary selected anatomical points or region of interest. Landmarks and regions of interest used for registration should fulfill the basic requirement of being stable during growth and unaffected by bone modeling associated with orthodontic tooth movement [20].

Scholarly literature includes numerous studies that have used software packages to quantify aligner treatment effects by digital superimpositions [7–9, 24]. Does the software being employed for superimposition affect the findings in a study is a question that is still unanswered. While several studies have validated the reliability of individual software packages, there is a paucity of reports assessing whether different registration platforms yield comparable outcomes when measuring the same patient cohort. This gap is critical, since discrepancies may directly influence the interpretation of clear aligner treatment accuracy.

To our knowledge, this is the first agreement study comparing three widely used commercial registration software platforms on the same cohort of clear aligner patients. Previous studies validated single programs in isolation, but none directly assessed inter-software comparability.

Therefore, the aim of this study was to evaluate the agreement of three popular 3D digital model registration software packages to measure tooth movement in clear aligner patients. The null hypothesis was that there is no statistically significant agreement between the different digital model registration software packages in measuring the amount of tooth movement in six degrees of freedom.

## Materials and methods

### Study design

This diagnostic agreement study was conducted following a modification of the Guidelines for Reporting Reliability and Agreement Studies (GRRAS) where each software package was considered as a rater [28]. Ethical approval was obtained by the institutional review board at the Faculty of Dentistry, Alexandria University, Egypt (IRB: 00010556-IORG: 0008839) and informed consents were obtained from all participants whose scans were utilized. All procedures adhered to the ethical committee’s relevant guidelines and regulations, with all identifiable patient information anonymized prior to analysis. The minimum sample size was determined based on prior studies assessing the reliability of newly developed software for 3D tooth movement analysis [19, 29]. A total of 32 scans was considered sufficient for this agreement study[30], based on assumptions of full agreement among raters, significance level of 95% (α = 0.05), and statistical power (1-β) of 80% using G* Power version 3.1.9.4 software. The initial calculation indicated a need for 30 scans, which was increased to 32 to accommodate potential scan defects.

## Sample collection

The study sample comprised full-arch pre-treatment (T1) and progress (T2) maxillary and mandibular intraoral digital scans from adult patients undergoing clear aligner therapy (CAT). These scans were randomly selected from the archives of a single orthodontic clinic in Mumbai, India, which has over 15 years of experience with CAT. A randomized list of 32 scans was generated using Microsoft Excel from the available records. All scans were acquired using a TRIOS 3D intraoral scanner (3Shape, Copenhagen, Denmark) and exported in STL file format before being imported into the three software programs under investigation. The selected cohort included 32 patients, each presenting with a Little’s Irregularity Index ranging from 4 to 6 mm, which is classified as *moderate irregularity* according to Little’s Index. All teeth, excluding third molars, were assessed for three-dimensional angular and linear movements in both arches. Inclusion criteria required that patients be adults treated with CAT involving both arches, with complete, high-quality scans displaying a full complement of teeth, excluding third molars. Exclusion criteria encompassed cases involving permanent tooth extractions, teeth with surface anomalies, or scans showing soft-tissue lesions affecting the palate or the mucogingival junction (MGJ) of the mandibular arch. Eligible scans were anonymized by an independent investigator and subsequently imported into the three designated tooth measurement software programs for evaluation by the principal investigator. ^(25–27)^

## Procedure

The T1 (pre-treatment) and T2 (progress) models were exported into three distinct tooth measurement software programs for registration and subsequent 3D linear and angular analyses. The software packages evaluated were:

1. Geomagic (G): A semiautomatic best-fit registration software (Geomagic U.S., Research Triangle Park, NC), utilizing a landmark-based approach followed by regional global surface registration [31].
2. OrthoAnalyzer (O): An interactive surface-based registration software (3Shape Ortho System, Copenhagen, Denmark), employing a surface 3-point registration method [32].
3. eModel 9.0 “Compare” (C): An automatic best-fit registration software (Geodigm Corporation, Chanhassen, MN), applying fully automated surface-to-surface registration [24].

For each software, the following procedural steps were executed:

(1) Registration (2) Generation of coordinate systems (3) Measurement of tooth movements.

### Registration of the T1 and T2 models using the three software packages Figure 1 [25–27]

**Fig. 1:**
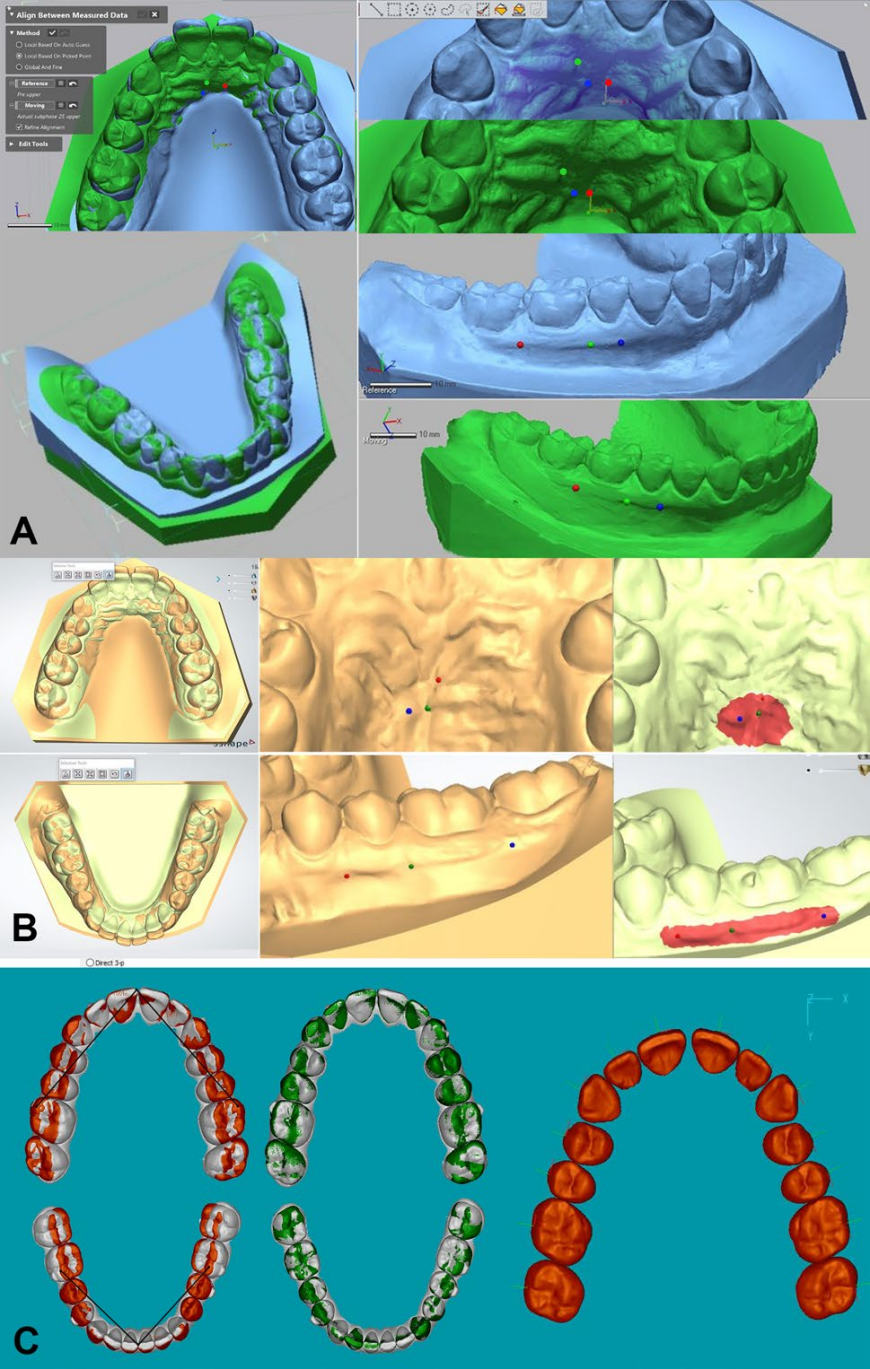
Maxillary and mandibular digital model superimpositions by the three tested software packages (**A**: Geomagic (G) (Geomagic U.S., Research Triangle Park, NC) software:https://www.engineering.pitt.edu/uploadedFiles/_Content/Sub_Sites/Business/MRW/SCPI/_Library/specs/geomagicdesignx2014userguide.pdf, **B**: OrthoAnalyzer (O) (3Shape Ortho System, Copenhagen, Denmark): http://promed.ua/wp-content/uploads/2012/01/2012_OrthoAnalyzer_English.pdf,2012, **C**: eModel 9.0 “Compare” (C)- (Geodigm Corporation, Chanhassen, MN)

#### Geomagic (Semiautomatic Best-Fit Registration)

Registration began with identifying stable anatomical landmarks on the palatal rugae and mucogingival junction (MGJ). This was followed by a global and fine regional surface alignment based on comprehensive point matching across both models.

#### OrthoAnalyzer (Interactive Surface-Based Registration)

This method involved selecting identical landmarks on corresponding models, complemented by marking a stable surface area to guide the surface-based registration.

#### Compare (Automatic Best-Fit Registration)

The process commenced with trimming and segmenting individual teeth on the T2 model. An initial global alignment was performed using three anatomical points—the mesial-buccal cusps of the first molars and the mesial-incisal edge of the right central incisor. This alignment was refined through 30 iterations of a closest-point algorithm to optimize occlusal surface fitting. Finally, an automated best-fit algorithm superimposed each segmented T2 tooth onto its counterpart in the unsegmented T1 model.

### Coordinate system generation

**Fig. 2:**
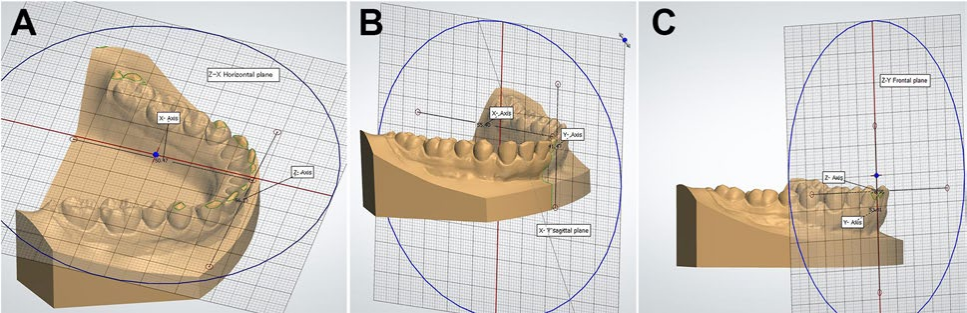
External reference planes used to measure differences in tooth movement between T1 and T2 (**A**: occlusal plane, **B**: sagittal plane, **C**: frontal plane)

Post registration, a three-dimensional (3D) coordinate system was established along the 3 principal axes to facilitate tooth movement measurements. Depending on the software used, either global model (Geomagic and OrthoAnalyzer software programs) or local tooth-specific frames (Compare software) were generated. Model global reference frames are defined as a coordinate system of three mutually perpendicular, intersecting axes (x = anteroposterior, y = occluso-gingival, and z = mediolateral). The “x-axis” is defined as the intersection of sagittal and occlusal planes, the “y-axis” as the intersection of the sagittal and coronal planes and the “z-axis” as the intersection of the coronal and occlusal planes [33]. The 3 D planes of space are the occlusal plane (XZ), midsagittal plane (XY), and the coronal plane (YZ). Figure 2.

Geomagic employed a single composite global coordinate system for the entire model with the three mutually perpendicular intersecting axes (X, Y, Z) and orthogonal planes were constructed (Composite Model Coordinates). On the other hand, OrthoAnalyzer required generating individual spatial reference frames for each tooth to individually measure tooth movements (Repeated Model Coordinates). Compare automatically created localized coordinate systems centered on each tooth (Automated Tooth Coordinates). Tooth positional changes were expressed as 3-vector translations and a 3 × 3 rotation matrix to capture movement in six degrees of freedom.

### 3D tooth movement measurements [25–27]

With T1 and T2 models aligned within a unified coordinate framework, tooth positional changes were quantified. The registration process produced a 3-vector translation and a 3 × 3 rotation matrix, capturing movement within a six-degrees-of-freedom system. Translational changes were recorded in millimeters—Buccolingual (BL), Mesiodistal (MD), and Occlusogingival (OG), while angular movements (Tip, Torque, and Rotation) were measured in degrees. Data from all three software programs were compiled and analyzed using Microsoft Excel (Microsoft Excel: 2016 Microsoft Corporation) for comparisons.

## Intra and inter-examiner reliability

All registrations, landmark identifications, axis definitions, and tooth movement measurements were initially performed by a single investigator (SA). To assess inter-operator reliability, a second calibrated examiner (NV) repeated measurements on eight randomly selected scan sets. After four weeks, SA re-evaluated another set of eight scans to determine intra-operator reliability. The collected data were pooled, and intraclass correlation coefficients (ICCs) were calculated to assess both intra- and inter-examiner consistency. ICCs were calculated using a two-way, fixed-rater, single-measure model with absolute agreement definition.

### Statistical analysis

Statistical analysis was carried out using IBM SPSS software package version 20.0. (Armonk, NY: IBM Corp). Data from individual teeth were pooled to provide an overall estimate of the amount of tooth movement in each degree of freedom and summarized as mean and standard deviation. Two-way, fixed-rater single-measure intraclass correlation coefficient (ICC) of absolute agreement were calculated between the pooled amount of tooth movement in each degree of freedom for each two software programs: Geomagic versus Compare (GC), Geomagic versus OrthoAnalyzer (GO) and Compare versus OrthoAnalyzer (CO). The data was found to be abnormally distributed. Based on the 95% confident interval of the ICC estimate, values less than 0.5, between 0.5 and 0.75, between 0.75 and 0.9, and greater than 0.90 are indicative of poor, moderate, good, and excellent reliability, respectively [34]. Statistical significance of the obtained results was expressed at *p* ≤ 0.01 to account for multiple comparisons.

## Results

Excellent intra- and inter-examiner reliability for pooled data were found for Geomagic and Compare software programs (intra-examiner reliability: 0.937, 0.953 respectively and inter-examiner reliability: 0.911, 0.940 respectively), while OrthoAnalyzer software showed good intra- and inter-examiner reliabilities for all the procedures (0.892, 0.869).

**Fig. 3:**
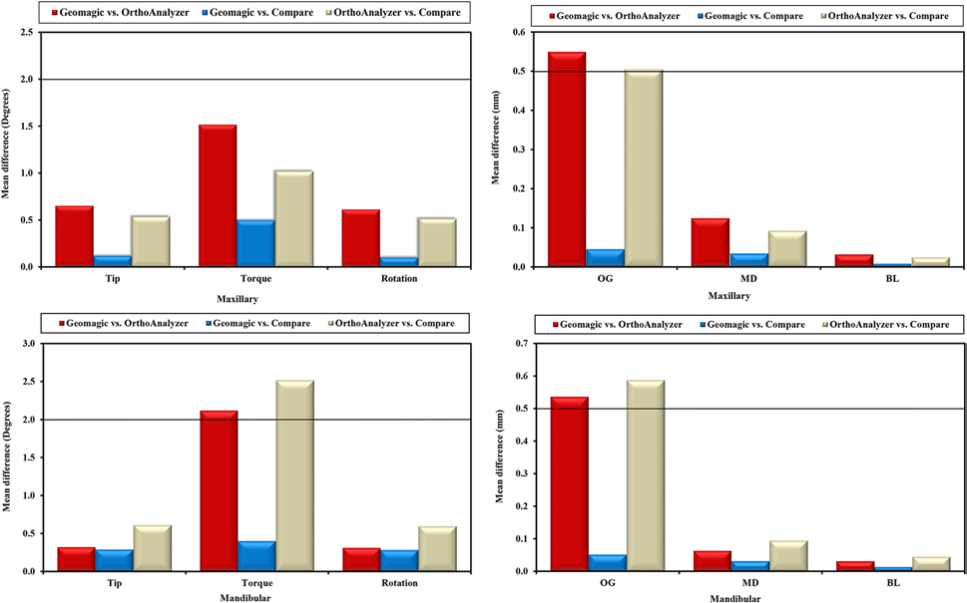
Bar graphs illustrating mean differences of maxillary and mandibular tooth movements in six degrees of freedom between each package pair against the clinical threshold

**Fig. 4:**
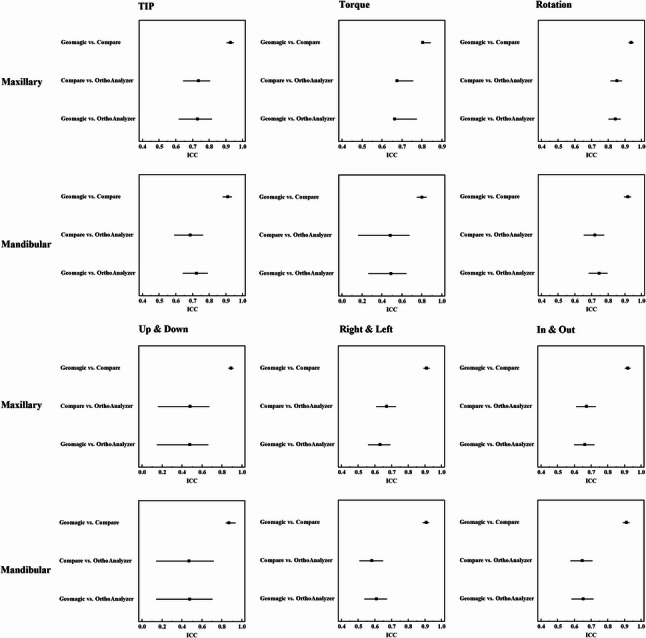
Forest plots demonstrating agreements between each two software packages presented as 95% confidence intervals for Intraclass Correlation Coefficients

Table 1 shows the descriptive statistics (mean and standard deviation) for the pooled maxillary and mandibular teeth with respect to the six angular and linear movements for the three tested softwares (Geomagic, OrthoAnalyzer and Compare). Figure 3 shows bar graphs illustrating the mean differences between each two software programs against a clinical threshold set at 0.5 mm for translations and 2º for angular measurements [3]. Agreements between each two software packages are presented as ICC in Table 2 and as forest plots in Figure 4.

**Table 1:** Pooled maxillary and mandibular tooth movement in six degrees of freedom determined by each software package

Type	Movements	No.	Geomagic	Orthoanalyzer	Compare
Maxillary	Tip	149	1.10 ± 4.50	0.45 ± 1.81	0.99 ± 3.42
	Torque	293	2.78 ± 4.22	1.26 ± 3.60	2.28 ± 3.50
	Rotation	238	1.29 ± 9.30	0.69 ± 7.50	1.20 ± 8.53
	OG	258	0.56 ± 0.81	0.01 ± 0.43	0.52 ± 0.73
	MD	351	0.17 ± 0.90	0.04 ± 0.51	0.14 ± 0.70
	BL	332	0.07 ± 1.24	0.03 ± 1.10	0.06 ± 1.11
Mandibular	Tip	155	0.34 ± 3.32	0.02 ± 2.54	0.63 ± 4.14
	Torque	215	0.61 ± 3.70	2.72 ± 4.30	0.21 ± 3.33
	Rotation	237	0.45 ± 9.01	0.14 ± 8.10	0.73 ± 10.71
	OG	267	0.81 ± 0.80	0.27 ± 0.52	0.86 ± 0.80
	MD	331	0.22 ± 1.01	0.16 ± 0.71	0.25 ± 1.11
	BL	294	0.04 ± 1.13	0.01 ± 1.01	0.06 ± 1.20

Data was expressed using Mean ± SD

**Table 2:** Intraclass correlation coefficients between each pair of software packages in six degrees of freedom for pooled maxillary and mandibular tooth movements

Movements	Type	Geomagic vs. Compare		Compare vs. Orthoanalyzer		Geomagic vs. Orthoanalyzer	
		ICC	95% C.I	ICC	95% C.I	ICC	95% C.I
TIP	Maxillary	0.929*	0.904–0.948	0.736*	0.643–0.806	0.730*	0.618–0.818
	Mandibular	0.912*	0.881–0.936	0.688*	0.591–0.765	0.725*	0.641–0.792
Torque	Maxillary	0.805*	0.757–0.844	0.675*	0.562–0.756	0.664*	0.477–0.774
	Mandibular	0.803*	0.749–0.847	0.484*	0.161–0.675	0.493*	0.265–0.646
Rotation	Maxillary	0.939*	0.922–0.953	0.853*	0.814–0.884	0.843*	0.802–0.876
	Mandibular	0.918*	0.895–0.936	0.721*	0.654–0.777	0.746*	0.683–0.797
OG	Maxillary	0.892*	0.864–0.915	0.483*	0.159–0.674	0.480*	0.148–0.664
	Mandibular	0.870*	0.838–0.938	0.473*	0.143–0.720	0.477*	0.141–0.707
MD	Maxillary	0.911*	0.892–0.928	0.671*	0.607–0.726	0.631*	0.558–0.693
	Mandibular	0.908*	0.887–0.925	0.582*	0.506–0.649	0.610*	0.537–0.673
BL	Maxillary	0.921*	0.902–0.936	0.673*	0.609–0.728	0.663*	0.599–0.720
	Mandibular	0.912*	0.891–0.930	0.648*	0.576–0.709	0.654*	0.583–0.715

Values less than 0.5, between 0.5 and 0.75, between 0.75 and 0.9, and greater than 0.90 are indicative of poor, moderate, good, and excellent reliability, respectively

*ICC* Intra class Correlation coefficient, *CI* Confidence interval, *LL* Lower limit, *UL* Upper Limit

*: Statistically significant at *p* ≤ 0.01

For Geomagic versus Compare, all differences in angular and linear movements were below the clinical threshold. On the other hand, for OrthoAnalyzer versus both Geomagic and Compare, the mandibular torque (2.17º, 2.52º respectively), as well as the maxillary OG movements (0.55 mm, 0.51 mm respectively) and mandibular OG movements (0.54 mm, 0.59 mm respectively) showed differences exceeding the clinical threshold.

Excellent agreements were found for all movements between Geomagic and Compare group, except for good agreements for torque and OG movements. On the contrary, the agreement between OrthoAnalyzer and both Geomagic and Compare showed a similar overall pattern. Maxillary rotations were the only movements with good agreements, moderate agreements were reported for all other movements except for poor agreements for mandibular torque (0.493, 0.484 respectively) and maxillary OG (0.480, 0.483 respectively) and mandibular OG movements (0.477, 0.473 respectively). All maxillary movements showed higher agreements than their mandibular equivalents which was consistent for the agreement between each pair. Torque and OG movements showed lowest agreements. Geomagic when compared to OrthoAnalyzer revealed lower agreements for all maxillary measurements in comparison to Compare with OrthoAnalyzer. The opposite was true for mandibular measurements.

## Discussion

CAT is one of the most robust applications of digital technology, where tooth movement is programmed to a simulation. When teeth are assigned a target position through virtual planning, quantifying their movements through the treatment process becomes integral to monitoring, tracking and planning efficacious therapy. Concurrent technology allows for the establishment of precise treatment goals and mechanics before treatment. “End in mind” driven orthodontic techniques have been studied extensively to know how close the final treatment results are to the planned corrections [7, 9, 24, 35]. To the best of our knowledge, this is the first study to evaluate agreement across three widely used commercial registration software platforms within the same cohort of clear aligner patients. While earlier research has validated individual programs separately, no prior work has directly compared inter-software performance.

Haouili [9] in 2020, reported an improvement in the overall accuracy of CAT, compared to Kravitz prospective trial in 2009[10].They reported a mean accuracy of 50% for all tooth movements, compared to the 41% accuracy in the 2009 trials, the treating clinician being the same. One difference between the two studies is the measurement software employed. Different studies reporting effectiveness of CAT have used various AI digital model registration programs, employing different algorithms and 3D measurements tools, to quantify the amounts of tooth movements [7–9, 11]. These software programs, however, have not been tested for agreement in measuring different movements of maxillary and mandibular teeth. The present study is the first agreement study to assess the agreement of three of the most commonly used software packages in the scholarly literature for the aforementioned purposes. The presented agreement study followed a modification of the Guidelines for Reporting Reliability and Agreement Studies (GRRAS) [28]. Adult patients undergoing clear aligner therapy without extraction were only included in the present study to exclude the effect of growth and treatment on the interpretation of results.

Since the software packages allow for the detection of differences that were too small to be clinically relevant, threshold values were chosen in reference to the American Board of Orthodontics (ABO) model grading system for case evaluation [36]. According to the model grading system criteria, discrepancies of 0.5 mm or greater in the alignment of contact points and marginal ridges will result in the deduction of points. A marginal ridge discrepancy of 0.5 mm equates to a crown-tip deviation of 2º for an average-sized molar. Therefore, differences of 0.5 mm or more in the MD, BL, and OG directions and differences of 2 º or more in tip, torque, and rotation were considered clinically relevant. Figure 3.

The restricted reference area in the maxilla was selected to ensure high precision and reproducibility in our superimposition methodology. While we acknowledge that wider areas have been validated in the literature, our choice was driven by the need to focus on regions that remain minimally affected by growth or orthodontic treatment, as suggested by Amir H. Abdi and M.Nouri in 2016. Wider areas may include structures that are prone to remodeling or variability during treatment, which could introduce inconsistencies. Therefore, medial and posterior landmarks on the rugae area were selected for the current study [37]. As for the mandibular superimposition, the selected MGJ area was used based on a study by Ioshida in 2019 comparing accuracy and reliability of mandibular digital model registration with use of the mucogingival junction as the reference. This study concluded that the method of DM registration of the mandible with use of the mucogingival junction as the reference is accurate, reliable, and reproducible [38]. 

Geomagic versus Compare group showed the highest agreements among all movements in comparison to other groups. Both Geomagic and Compare use a best fit surface algorithm. This technique of fine automatic matching uses thousands of reference points instead of a few landmarks/areas and is based on ‘iterative closest point algorithms’[39], improving the quality of superimposition by consecutive iterations. Though a best fit algorithm was used in both Geomagic and Compare software programs, they demand different landmarks identification before proceeding automatically. Geomagic software used anatomical landmarks on the rugae and MGJ areas that have been considered stable [18, 38, 40]. On the contrary, Compare used landmarks on molars and incisors for initial alignment.

Differently, lower agreements were calculated when OrthoAnalyzer was compared to each of the other software programs across all movements. This can be attributed to the algorithm mandated by the software, which involves selection of only a specified area/few landmarks of the surface increasing sources of potential errors. The algorithm also doesn’t iterate to improve the accuracy of matchings, accounting for the lower agreements. Garib et al.[41] and Ganzer et al.[20] reported results in accordance with the present study, where they found that the definition of a specified region of interest is dependent on the same landmark identification used for point of interest registration. Therefore, errors in the identification landmark might decrease the reliability of both registration methods. Furthermore, manual selection of individual landmarks and measurement of matchings can be a source of error, since small errors in detecting the coordinates of the landmark points manifest as significant errors in the resulting alignment.

Additionally, the higher agreements in Geomagic versus Compare group could be explained by another critical factor dictating the overall accuracy of tooth movements measurements which is the generation of reproducible coordinate system. Geomagic uses one global model coordinate system and Compare software generates automated computations for placement of local tooth coordinate systems at each tooth’s approximate centre of resistance [7, 24, 42]. Contrastingly, OrthoAnalyzer software requires the generation of multiple customized model global coordinates for each tooth measured. This, undoubtedly, introduced more operator error each time a plane or an axis was generated.

Rotations had the highest agreements among angular movements between all pairs of software programs. This could be ascribed to the use of external occlusal planes instead of using internal long axes for rotation measurements. Our findings were similar to those reported by Chong et al. [43] who used an external reference plane. Grauer et al.[42] found rotations to have the largest discrepancies due to measurements along the long axes of teeth and not along an external reference plane. Figure 2 Torque ICC values were the least amongst all angular measurements between all pairs of software programs. This could be explained by the inaccuracy encountered during the placement of tangents on the labial surfaces, especially on more convex teeth. The mandibular torque was the only angular movement that showed poor agreements and exceeded the 2º clinical threshold in the two groups involving OrthoAnalyzer.

The present study also found that mandibular agreements were always less than maxillary equivalents for all movements, among all tested groups. These findings can be attributed to the landmarks used for mandibular registration. These have been tested for reliability in two studies [38, 44]. This implies that the mandibular superimposition using the MGJ landmarks as a reference is less accurate than the maxillary superimposition using the rugae area. Although the mucogingival line is a stable anatomic landmark that is not permanently altered by either orthodontics or surgery [45], the validated methods used in the study by Ioshida et al.[38] may have greater errors if teeth have been moved out of the alveolar bone (i.e., when an alveolar bone dehiscence is created) or if severe periodontal disease develops longitudinally. They added that the main limitations of this methodology might become evident if therapy includes a large amount of tooth movement (i.e., orthodontic expansion or a great amount of extrusion) [46] and signs of an inflammatory response in the gingival area.

Occlusogingival movements of the maxillary and mandibular teeth (Intrusion/Extrusion) were the only linear movements revealing poor agreements, as well as exceeding the 0.5 mm clinical threshold in Geomagic versus OrthoAnalyzer and Compare versus Ortho-Analyzer groups. Using metallic implants, Gu and McNamara [47] have found that the lingual curvature of the hard palate shows apposition and downward displacement during growth. It was assumed by Garib et al. [41] that palatal rugae follow hard palate changes. Therefore, the superimposition on the palatine rugae might under-quantify the vertical movements of the maxillary arch. This underestimation is a potential drawback of registering digital dental models on the palatal rugae. An et al. [44] found that the difference in mandibular vertical incisor movement was larger than the difference in horizontal movement. They further illustrated that errors along the y-axis may be more substantial than those in the x-axis because of the morphological characteristics of alveolar bone, namely its rather flat, vertically oriented surfaces which more or less fit into each other in different vertical positions. Because of this reason, researchers and clinicians should cautiously interpret vertical measurements relative to structures of reference used.

Linear agreements consistently showed lower agreements than maxillary ones for the same movements in all three groups. This could be ascribed to the landmarks used as a reference for measurements. Angular measurements depended on projection of pre and post long axes on corresponding planes. On the other hand, linear measurements in Geomagic and OrthoAnalyzer software programs utilized incisal edges and cusp tips as reference points. Since the occlusal third including the incisal edge or cusp tip showed greater anatomic variations than the gingival third and could be changed during treatment because of attrition, fracture, or reshaping, it might be confounding to measure tooth movement. The facial axis point, however, is easily recognized and used as a relatively stable guide for bracket positioning. In addition, the incisal edge and cusp tip can have greater changes due to the longer distance from the center of rotation compared to the FA point [48]. Facial axis point was not applicable by these two software programs for pre and post measurements of points. Compare software, on the contrary, was the only one using local coordinates placed at tooth’s centroid for measurements. Therefore, linear measurements by this software were the most representative of the whole tooth movement. The software calculates differences automatically and is, therefore, less prone to bias. However, the arduous process of model preparation and segmentation may introduce some variations.

Overall, lower agreements with OrthoAnalyzer can be attributed to its reliance on manually selected landmarks and limited surface areas, which are operator-dependent and do not employ iterative refinement. By contrast, Geomagic and Compare employ iterative closest point surface algorithms, using thousands of points to minimize error. Landmark dependency particularly compromises mandibular measurements, where mucogingival junction landmarks are less stable.

Clinical implications include that torque measurements, particularly in mandibular teeth, showed the lowest agreements and exceeded the 2º clinical threshold in groups involving OrthoAnalyzer. Such discrepancies could mislead clinicians when evaluating the predictability of aligner therapy. Similarly, occlusogingival discrepancies above 0.5 mm suggest that vertical outcomes should be interpreted cautiously, especially in extraction or extrusion cases. Furthermore, mandibular torque and maxillary/mandibular OG directions involving OrthoAnalyzer are not suitable for inter-software interchangeable use, and this directly impact clinical decision-making, including precise control of root torque and attachment design selection when mandibular torque is indicated.

The clinical relevance of these findings lies in the careful selection of digital tools for outcome assessment in clear aligner therapy. Based on the results, OrthoAnalyzer demonstrated poor reliability in mandibular torque measurements (Rx) and vertical displacements (Z-axis) across multiple software pairings. This suggests that OrthoAnalyzer should not be used interchangeably with Geomagic or Compare for assessing these particular dimensions. In contrast, Geomagic and Compare exhibited excellent consistency, particularly for tip (Ry) and transverse (X) measurements in both arches. Clinicians and researchers are advised to select software based on the specific dimensional priorities of their analysis. For instance, if the goal is to evaluate torque or vertical control in lower anterior teeth, Compare or Geomagic may provide more reproducible results. Conversely, if only angular tip measurements are needed, any of the three platforms may suffice, though variability in OrthoAnalyzer should still be considered. These software-specific recommendations aim to enhance the reliability and clinical utility of digital outcome assessments in orthodontic practice.

Limitations of the present study include the fact that registration of digital models allows assessments of individual tooth movement rather than overall skeletal displacement of the craniofacial complex, as registration relative to the cranial base allows. Also, all measurements are based on crowns without referring to roots. Given the relatively higher radiation exposure and current health regulations, three- dimensional analyses using CBCT purely for treatment analyses are not ethically justified for tooth movement calculations [49]. Future investigations warranted are studying percentage accuracy of the different tooth movements based on superimpositions of treated outcomes with simulations using different software packages with high agreements.

Although this study provides valuable insight into the inter-software consistency of three commonly used 3D registration platforms, it is important to emphasize that the absence of a true reference standard (e.g., CBCT-based measurements or standardized in vitro models) using implant-based reference points) prevents any conclusion about absolute measurement accuracy. Without external validation against a known “ground truth,” our data reflect relative agreement between platforms, not correctness. Clinically, this implies that even if two platforms appear consistent, they may both deviate systematically from true tooth positions. As such, the findings should be interpreted cautiously when used to inform treatment decisions or outcome evaluations, especially in cases requiring high precision such as root torque assessment or vertical intrusion/extrusion. Future studies incorporating reference-validated imaging modalities are needed to confirm the clinical accuracy of these software tools.

## Conclusions

1. Compare and Geomagic software programs showed the highest agreements with each other, for all movements in comparison to the other groups evaluated.
2. Torque and occluso-gingival movements (intrusion/extrusion) were the only movements with poor agreements, exceeding the clinical threshold in the groups involving OrthoAnalyzer software.
3. The difference between the tooth movement measured by Geomagic and Compare was not clinically significant.

## Data Availability

The datasets used and/or analyzed during the current study are available in the manuscript.

## Abbreviations

ABO: American board of orthodontics
AI: Artificial intelligence
BL: Buccolingual
CAD/CAM: Computer-aided design / computer-aided manufacturing
CAT: Clear aligner therapy
CBCT: Cone-beam computed tomography
CR: Centre of resistance
FA Point: Facial axis point
GRRAS: Guidelines for reporting reliability and agreement studies
ICC: Intraclass correlation coefficient
IRB: Institutional review board
MD: Mesiodistal
MGJ: Mucogingival junction
OG: Occlusogingival
ROI: Region of interest
STL: Standard tessellation language (3D file format)
T1: Pre-treatment scan/time point 1
T2: Progress scan/time point 2
3D: Three-dimensional

## References

[1] Rossini G, Parrini S, Castroflorio T, Deregibus A, Debernardi CL. Diagnostic accuracy and measurement sensitivity of digital models for orthodontic purposes: A systematic review. Am J Orthod Dentofac Orthop. 2016;149:161–70. 10.1016/j.ajodo.2015.06.029. 10.1016/j.ajodo.2015.06.029 26827972

[2] De Luca Canto G, Pachêco-Pereira C, Lagravere MO, Flores-Mir C, Major PW. Intra-arch dimensional measurement validity of laser-scanned digital dental models compared with the original plaster models: a systematic review. Orthod Craniofac Res. 2015;18:65–76. 10.1111/ocr.12068. 25677755 10.1111/ocr.12068

[3] AlSeraidi M, Hansa I, Dhaval F, Ferguson DJ, Vaid NR. The effect of vestibular, lingual, and aligner appliances on the quality of life of adult patients during the initial stages of orthodontic treatment. Prog Orthod. 2021;22:3. 10.1186/s40510-020-00346-0. 33458787 10.1186/s40510-020-00346-0 PMC7811964

[4] Kravitz ND, Hansa I, Vaid NR, Moshiri M, Adel SM. Does age influence deep overbite correction with Invisalign? A prospective study evaluating mandibular incisor intrusion in adolescents vs adults. Angle Orthod. 2024;94:145–50. 10.2319/050223-320.1. 37939782 10.2319/050223-320.1 PMC10893929

[5] Adel SM, Bichu YM, Pandian SM, Sabouni W, Shah C, Vaiid N. Clinical audit of an artificial intelligence (AI) empowered smile simulation system: a prospective clinical trial. Sci Rep. 2024;14:19385. 10.1038/s41598-024-69314-6. 39169095 10.1038/s41598-024-69314-6 PMC11339289

[6] Adel S, Hansa I, Vaid N. Clear aligner therapy in contemporary orthodontics: a scoping review of scholarly literature. APOS Trends Orthod. 2023;14:3–27. 10.25259/APOS_215_2022.

[7] Grünheid T, Loh C, Larson BE. How accurate is Invisalign in nonextraction cases? Are predicted tooth positions achieved? Angle Orthod. 2017;87:809–15. 10.2319/022717-147.1. 28686090 10.2319/022717-147.1 PMC8317555

[8] Sachdev S, Tantidhnazet S, Saengfai NN. Accuracy of tooth movement with in-house clear aligners. J World Fed Orthod. 2021;10:177–82. 10.1016/j.ejwf.2021.08.003. 34625386 10.1016/j.ejwf.2021.08.003

[9] Haouili N, Kravitz ND, Vaid NR, Ferguson DJ, Makki L. Has Invisalign improved? A prospective follow-up study on the efficacy of tooth movement with Invisalign. Am J Orthod Dentofacial Orthop. 2020;158:420–5. 10.1016/j.ajodo.2019.12.015. 32620479 10.1016/j.ajodo.2019.12.015

[10] Kravitz ND, Kusnoto B, BeGole E, Obrez A, Agran B. How well does Invisalign work? A prospective clinical study evaluating the efficacy of tooth movement with Invisalign. Am J Orthod Dentofacial Orthop. 2009;135:27–35. 10.1016/j.ajodo.2007.05.018. 19121497 10.1016/j.ajodo.2007.05.018

[11] Dai FF, Xu TM, Shu G. Comparison of achieved and predicted tooth movement of maxillary first molars and central incisors: first premolar extraction treatment with Invisalign. Angle Orthod. 2019;89:679–87. 10.2319/090418-646.1. 30920875 10.2319/090418-646.1 PMC8111827

[12] Alwafi A, Bichu YM, Avanessian A, Adel SM, Vaid NR, Zou B. Overview of systematic reviews and meta-analyses assessing the predictability and clinical effectiveness of clear aligner therapy. Dent Rev. 2023;3:100074. 10.1016/j.dentre.2023.100074.

[13] Stucki S, Gkantidis N. Assessment of techniques used for superimposition of maxillary and mandibular 3D surface models to evaluate tooth movement: a systematic review. Eur J Orthod. 2020;42:559–70. http://doi10.1093/ejo/cjz075. 31742598 10.1093/ejo/cjz075

[14] Vaid NR. Digital technologies in orthodontics 2013; an update. Semin Orthod. 2018;24:373–5. 10.1053/j.sodo.2018.10.001.

[15] Bichu YM, Weir T, Zou B, Adel S, Vaid NR. Clear aligner therapy concerns: addressing discrepancies between digitally anticipated outcomes and clinical ground realities. Turk J Orthod. 2024;37:130–9. http://doi10.4274/TurkJOrthod.2024.2024.4. 38952301 10.4274/TurkJOrthod.2024.2024.4

[16] Vaid NR, Adel SM. Semin. Orthod. Elsevier. 2023. pp. 1–3.

[17] Vaid NR, Sabouni W, Wilmes B, Bichu YM, Thakkar DP, Adel SM. Customized adjuncts with clear aligner therapy: the golden circle model explained! J World Fed Orthod. 2022;11:216–25. http://doi10.1016/j.ejwf.2022.10.005. 36400659 10.1016/j.ejwf.2022.10.005

[18] Vasilakos G, Schilling R, Halazonetis D, Gkantidis N. Assessment of different techniques for 3D superimposition of serial digital maxillary dental casts on palatal structures. Sci Rep. 2017;7(5838). http://doi10.1038/s41598-017-06013-5. 10.1038/s41598-017-06013-5 PMC5517608 28724930

[19] Talaat S, Kaboudan A, Bourauel C, Ragy N, Kula K, Ghoneima A. Validity and reliability of three-dimensional palatal superimposition of digital dental models. Eur J Orthod. 2017;39:365–70. http://doi10.1093/ejo/cjx008. 28339627 10.1093/ejo/cjx008

[20] Ganzer N, Feldmann I, Liv P, Bondemark L. A novel method for superimposition and measurements on maxillary digital 3D models-studies on validity and reliability. Eur J Orthod. 2018;40:45–51. http://doi10.1093/ejo/cjx029. 28444179 10.1093/ejo/cjx029

[21] Bichu YM, Hansa I, Bichu AY, Premjani P, Flores-Mir C, Vaid NR. Applications of artificial intelligence and machine learning in orthodontics: a scoping review. Prog Orthod. 2021;22:18. 10.1186/s40510-021-00361-9. 34219198 10.1186/s40510-021-00361-9 PMC8255249

[22] Vaid NR. Semin. Orthod. Elsevier. 2021. pp. 57–61.

[23] Oliveira FP, Tavares JM. Medical image registration: a review. Comput. Methods. Biomech. Biomed. Engin. 2014;17:73–93. 10.1080/10255842.2012.670855. 10.1080/10255842.2012.670855 22435355

[24] Awad MG, Ellouze S, Ashley S, Vaid N, Makki L, Ferguson DJ. Semin. Orthod. Elsevier. 2018. pp. 393–406.

[25] Adel SM, Vaid NR, El-Harouni N, Kassem H, Zaher AR. TIP, TORQUE & ROTATIONS: how accurately do digital superimposition software packages quantify tooth movement? Prog Orthod. 2022;23(8). 10.1186/s40510-022-00402-x. 10.1186/s40510-022-00402-x PMC8918442 35284950

[26] Adel SM, Vaid NR, El-Harouni N, Kassem H, Zaher AR. Digital model superimpositions: are different software algorithms equally accurate in quantifying linear tooth movements? BMC Oral Health. 2022;22(103). 10.1186/s12903-022-02129-x. 10.1186/s12903-022-02129-x PMC8973572 35361187

[27] Adel SM, Vaid NR, El-Harouni N, Kassem H, Park JH, Zaher AR. Quantifying maxillary anterior tooth movement in digital orthodontics: does the choice of the superimposition software matter? J World Fed Orthod. 2023;12:187–96. http://doi10.1016/j.ejwf.2023.07.002. 37625927 10.1016/j.ejwf.2023.07.002

[28] Kottner J, Audigé L, Brorson S, Donner A, Gajewski BJ, Hróbjartsson A, Roberts C, Shoukri M, Streiner DL. Guidelines for Reporting Reliability and Agreement Studies (GRRAS) were proposed. J. Clin. Epidemiol. 2011;64:96–106. 10.1016/j.jclinepi.2010.03.002. 10.1016/j.jclinepi.2010.03.002 21130355

[29] Talaat S, Kaboudan A, Breuning H, Ragy N, Elshebiny T, Kula K, Ghoneima A. Reliability of linear and angular dental measurements with the orthomechanics sequential analyzer. Am J Orthod Dentofac Orthop. 2015;147:264–9. http://doi10.1016/j.ajodo.2014.07.027. 10.1016/j.ajodo.2014.07.027 25636561

[30] Z., L. J. J. Sample size calculation for an agreement study. (2010). Pharm Stat 9, 125–32. 10.1002/pst.382. 10.1002/pst.382 19507134

[31] Geomagic. Geomagic design X user guide. (2013). Available at: https://www.engineering.pitt.edu/uploadedFiles/_Content/Sub_Sites/Business/MRW/SCPI/_Library/specs/geomagicdesignx2014userguide.pdf.

[32] 3 Shape Ortho System. OrthoAnalyzer 2012 User Manual. (2012). Available at: https://promed.ua/wp-content/uploads/2012/01/2012_OrthoAnalyzer_English.pdf,2012.

[33] Daskalogiannakis J. Glossary of orthodontic terms. Chicago: Quintessence Pub. Co; 2000.

[34] Koo TK, Li MY. A guideline of selecting and reporting intraclass correlation coefficients for reliability research. J Chiropr Med. 2016;15:155–63. 10.1016/j.jcm.2016.02.012. 27330520 10.1016/j.jcm.2016.02.012 PMC4913118

[35] Mortazavi M, Naeim M, Badri A, Sharifi R, Hasheminasab M. An updated systematic review on the effectivity of clear aligner therapy: a review. J Craniomaxillofac Res. 2020;165:165–77. 10.18502/jcr.v7i4.5552.

[36] American Board of Orthodontics. Grading system for dental casts and panoramic radiographs. Available at: https://www.americanboardortho.com/media/1191/grading-system-castsradiographs.pdf. 10.1016/s0889-5406(98)70179-9 9810056

[37] Abdi AH, Nouri M. Registration of serial maxillary models via the weighted rugae superimposition method. Orthod. Craniofac. Res. 2017;20:79–84. 10.1111/ocr.12142. 10.1111/ocr.12142 28150411

[38] Ioshida M, Muñoz BA, Rios H, Cevidanes L, Aristizabal JF, Rey D, Kim-Berman H, Yatabe M, Benavides E, Alvarez MA, Volk S, Ruellas AC. Accuracy and reliability of mandibular digital model registration with use of the mucogingival junction as the reference. Oral. Surg. Oral. Med. Oral. Pathol. Oral. Radiol. 2019;127:351–360. 10.1016/j.oooo.2018.10.003. 10.1016/j.oooo.2018.10.003 30472195

[39] Pomerleau F, Colas F, Siegwart R, Magnenat S. Comparing ICP variants on real-world data sets: open-source library and experimental protocol. Auton Robot. 2013;34:133–48. 10.1007/s10514-013-9327-2.

[40] Chen G, Chen S, Zhang XY, Jiang RP, Liu Y, Shi FH, Xu TM. Stable region for maxillary dental cast superimposition in adults, studied with the aid of stable miniscrews. Orthod. Craniofac. Res. 2011;14:70–79. 10.1111/j.1601-6343.2011.01510.x. 10.1111/j.1601-6343.2011.01510.x 21457456

[41] Garib D, Miranda F, Yatabe MS, Lauris JRP, Massaro C, McNamara JA Jr., et al. Superimposition of maxillary digital models using the palatal rugae: does ageing affect the reliability? Orthod Craniofac Res. 2019;22:183–93. 10.1111/ocr.12309. 30844126 10.1111/ocr.12309 PMC6642031

[42] Grauer D, Proffit WR. Accuracy in tooth positioning with a fully customized lingual orthodontic appliance. Am. J. Orthod. Dentofacial. Orthop. 2011:140:433–443. 10.1016/j.ajodo.2011.01.020. 10.1016/j.ajodo.2011.01.020 21889089

[43] Chong D-R, Jang Y-J, Chun Y-S, Jung S-H, Lee S-K. The evaluation of rotational movements of maxillary posterior teeth using three dimensional images in cases of extraction of maxillary first premolar. Korean J Orthod. 2005;451–8.

[44] An K, Jang I, Choi DS, Jost-Brinkmann PG, Cha BK. Identification of a stable reference area for superimposing mandibular digital models. J Orofac Orthop. 2015;76:508–19. http://doi10.1007/s00056-015-0310-8. 26250456 10.1007/s00056-015-0310-8

[45] Wennström JL. Mucogingival considerations in orthodontic treatment. (1996). Semin. Orthod. 2: 46–54. 10.1016/s1073-8746(96)80039-9. 10.1016/s1073-8746(96)80039-9 9161283

[46] Pikdoken L, Erkan M, Usumez S. Gingival response to mandibular incisor extrusion. (2009). Am. J. Orthod. Dentofacial. Orthop. 2009;135:432.e431-436; discussion 432–433. 10.1016/j.ajodo.2009.01.005. 10.1016/j.ajodo.2009.01.005 19361726

[47] Gu Y, McNamara JA Jr. Cephalometric superimpositions. Angle. Orthod. 2008;78:967–976. 10.2319/070107-301.1. 10.2319/070107-301.1 18947269

[48] Smith RN, Karmo M, Russell J, Brook AH. The variability of the curvature of the labial surface of the upper anterior teeth along the facial axis of the clinical crown. Arch. Oral. Biol. 2007;52:1037–1042. 10.1016/j.archoralbio.2007.04.017. 10.1016/j.archoralbio.2007.04.017 17617372

[49] Choi DS, Jeong YM, Jang I, Jost-Brinkmann PG, Cha BK. Accuracy and reliability of palatal superimposition of three-dimensional digital models. Angle. Orthod. 2010;80:497–503. 10.2319/101309-569.1. 10.2319/101309-569.1 PMC8966451 20482354

